# High concentration of coagulase-negative staphylococci carriage among bioaerosols of henhouses in Central China

**DOI:** 10.1186/s12866-020-1709-y

**Published:** 2020-01-28

**Authors:** Yuanqing Lu, Qin Lu, Yiluo Cheng, Guoyuan Wen, Qingping Luo, Huabin Shao, Tengfei Zhang

**Affiliations:** Key laboratory of prevention and control agents for animal bacteriosis, Institute of Animal Husbandry and Veterinary, Hubei Academy of Agricultural Sciences, Wuhan, 430064 China

**Keywords:** Bioaerosol, Coagulase-negative staphylococci, Antibiotic resistance, *mecA*, Inflammatory cytokine

## Abstract

**Background:**

Coagulase-negative staphylococci (CoNS) are a group of opportunistic pathogens, which are widely spread in the environment. Animal breeding is an important source of pathogen spreading. However, the concentration and characteristics of CoNS in the bioaerosols of henhouses are unclear.

**Results:**

In this study, we showed that CoNS were significantly increased in bioaerosols of henhouses during the first 60 days, and reached 2.0 × 10^6^ CFU/m^3^, which account for 75.4% of total bacteria. One hundred and two CoNS isolates from bioaerosols and nasal swabs of farmers were further identified, covering seven species. Among these, 41.2% isolates were *Staphylococcus sciuri*, which was the predominant species, followed by *S. equorum*, *S. saprophyticus*, *S. haemolyticus*, *S. xylosus*, *S. arlettae* and *S. gallinarum*. There were high rates of resistance to oxacillin in CoNS (49.0%), which were defined as Methicillin-Resistant CoNS (MRCoNS), and 36.3% isolates contained resistance gene *mecA*. Bioaerosol infection models showed that, chickens exposed to aerosolized *S. sciuri* had significant induction of inflammatory cytokines interleukin (IL)-1β, IL-6, IL-8 and IL-10 at 5 days post-infection (dpi) in lungs and at 7 dpi in spleens.

**Conclusions:**

We reported a high concentration of CoNS in henhouses, and *S. sciuri* was the preponderant CoNS species. Antibiotic resistance analysis and bioaerosols infection of CoNS further highlighted its hazards on resistance and immunological challenge. These results suggested that, CoNS in bioaerosols could be one serious factor in the henhouses for not only poultry industry but also public health.

## Background

Coagulase-negative staphylococci (CoNS) are a group of opportunistic pathogens, which are not only in animals and humans but also widely spread in the environment, such as dust, soil, water and air [1–3]. CoNS can cause human and animal infections. In humans, CoNS are associated with endocarditis, septicemia and blood stream infection, and they have become one of the most important sources of hospital infection [4, 5]. In chickens, CoNS infection can cause arthritis, fibrinopurulent blepharitis and even systemic infection [6–8]. In addition, methicillin-resistant CoNS (MRCoNS)-contaminated chicken meat is frequently reported, suggesting foodborne transmission of the bacteria [9, 10].

Recently, the reports on multi-resistant CoNS were increasing, with the increase in antibiotic usage [11]. The increasing antibiotic resistance of CoNS also limits the drug choices for treatment of CoNS infections [12]. What is more, CoNS exist in the places where antibiotics are widely used, such as hospitals and animal farms [13, 14], which accelerate the spread of resistance genes. Methicillin-resistant *Staphylococcus aureus* (MRSA) and vancomycin-resistant *S. aureus* (VRSA) have been frequently reported [15, 16], and show similar resistance genes to CoNS, such as the methicillin-resistance gene *mecA* [17]. As a widespread bacterium in the environment, CoNS has been considered as a reservoir of resistance genes, which highlights its threat to public health.

Bioaerosols, mainly including bacteria, viruses and fungi, are potential environmental sources of animals and human infection. For animals, piglets exposed to 10^6^ cfu/m^3^ MRSA in the air were persistent colonized and 10^4^ cfu/m^3^ were transient [18]. MRCoNS carriage analysis suggested potential transmission of MRCoNS from livestock to humans by occupational livestock contact [19]. In poultry houses, bioaerosol is one important route of transmission of viral as well as bacterial pathogens. In Switzerland, an analysis on 12 poultry houses showed that the mean bacterial exposure level to poultry farmers was 53 × 10^7^ cells/m^3^, and among them, 62 × 10^6^ cells/m^3^ were staphylococci [20]. In Canada, *Enterococcus* spp*.*, *Escherichia coli*, and *Staphylococcus* spp*.* spread widely in bioaerosols of poultry houses, and high levels of zinc bacitracin, erythromycin and tetracycline resistance genes have been found in bioaerosols [21]. Therefore, bioaerosols in animal houses are potentially harmful to the health of both animals and farmers. In addition, besides livestock manure and waste water, bioaerosol is another important pathway for diffusing pathogens and resistance genes to the outside environment.

The goal of this study was to investigate the concentration and antibiotic resistance of CoNS in bioaerosols from henhouses in China, and further examine its effect on immune response of chickens. This study will provide important information for the poultry industry and public health.

## Results

### CoNS in bioaerosols and farmers

**Fig. 1:**
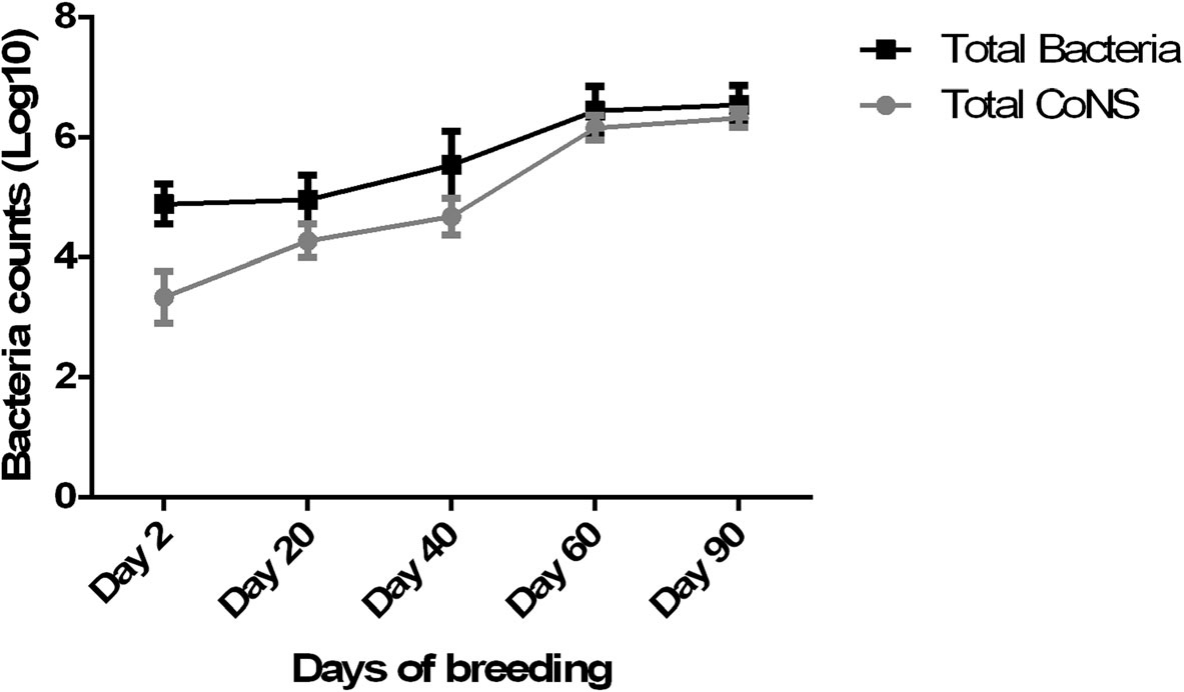
Increasing of total bacteria and CoNS during chicken breeding

Bioaerosol samples were collected from nine henhouses covered the first 90 days of the growing period before changing their cage. As shown in Fig. 1, at day 2, the mean total bacterial count was 7.8 × 10^4^ cfu/m^3^ in the bioaerosols of henhouses, and among them, the mean of CoNS was 1.7 × 10^3^ cfu/m^3^, which accounted for approximately 2.2% of total bacteria. As time progressed, the total bacterial count, especially the total CoNS count, was significantly increased during the first 60 days. At day 60, the mean total bacterial count was 2.6 × 10^6^ cfu/m^3^, which was approximately 34 -fold of that at day 2, and among them, 75.4% (2.0 × 10^6^ cfu/m^3^) of bacteria were CoNS. At day 90, the bacterial count was similar to that at day 60 (*p* > 0.05). These results suggested that CoNS was the primary genus in the bioaerosols of henhouses. In addition, nasal swabs from 14 poultry farmers were also evaluated, and among them, eight (57.1%) samples were CoNS-positive.

To characterize further the CoNS in bioaerosols and farmers, 102 CoNS were randomly picked and further identified. As shown in Table 1, 102 isolates covered seven species. Forty-two (41.2%) isolates were *S. sciuri*, which was the predominant species, followed by *S. equorum* (21.6%), *S. saprophyticus* (18.6%) and *S. haemolyticus* (10.8%). The others included *S. xylosus* (5.9%), *S. arlettae* (1.0%) and *S. gallinarum* (1.0%). Among them, five *S. sciuri*, two *S. equorum* and one *S. haemolyticus* isolates were from nasal swabs of farmers.

**Table 1:** The distribution of *mecA* in different species of CoNS strains

Species	No. of strains	Proportions in CoNS	No. of *mecA* positive strains	*mecA* Positive rates
*S. equorum*	22 (2)^a^	21.57%	4 (1)	18.18%
*S. haemolyticus*	11 (1)	10.78%	0	0.00%
*S. saprophyticus*	19	18.63%	0	0.00%
*S. sciuri*	42 (5)	41.18%	30 (2)	71.43%
*S. xylosus*	6	5.88%	2	33.33%
*S. arlettae*	1	0.98%	0	0.00%
*S. gallinarum*	1	0.98%	1	100.00%
Total	102 (8)	100.00%	37 (3)	36.27%

^a^The No. in the brackets indicate the number of isolates from the nasal swabs of farmers

### Presence of *mecA* and antibiotic resistance rates in CoNS

**Fig. 2:**
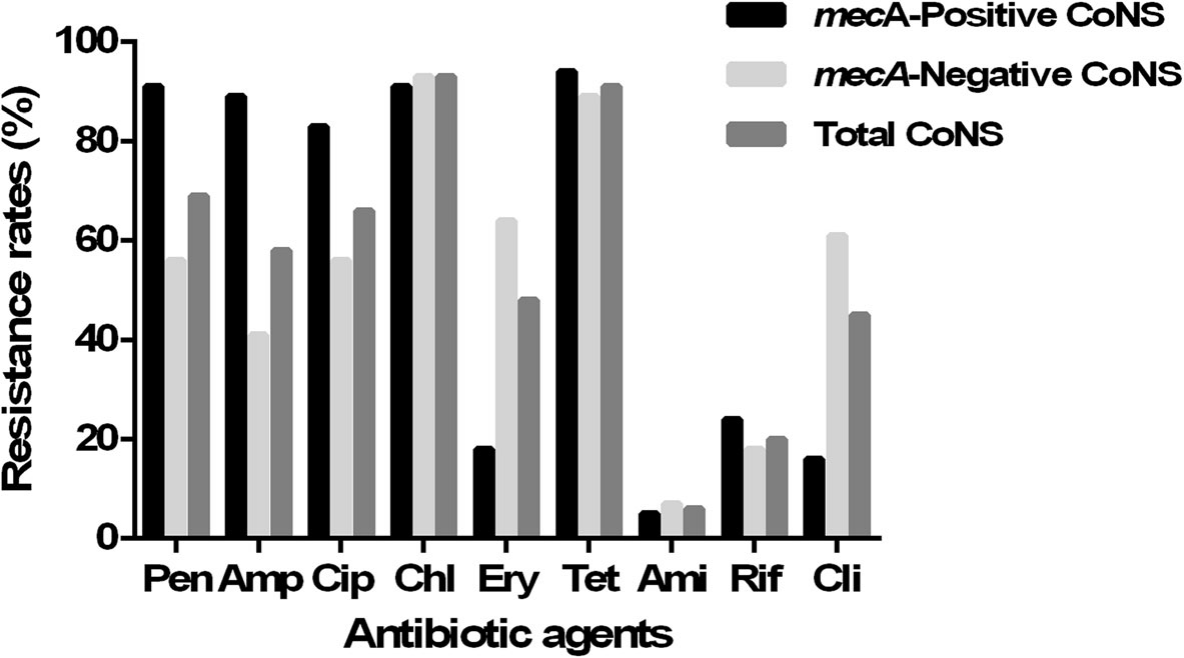
Antibiotic resistance of CoNS to nine drugs

The susceptibility of CoNS isolates to nine antibiotics was tested using disk diffusion assays (Fig. 2). The isolates showed high resistance rates to antibiotics that are widely used in animal breeding, including penicillin (69.61%), ampicillin (58.82%), ciprofloxacin (66.67%), chloramphenicol (93.14%), erythromycin (48.04%), tetracycline (91.18%) and clindamycin (45.10%). In contrast, lower resistance rates were seen for amikacin (6.86%) and rifampicin (20.59%), which are less used in breeding. Susceptibility to oxacillin and vancomycin, which are two important antibiotics used in human, were tested using MIC assays (Table 2). The MICs of oxacillin in 49.0% isolates were > 2 μg/ml, including all of the *mecA*-positive isolates and 20% (13/65) *mecA*-negative isolates. The MICs of vancomycin in 40.2% isolates were between 4 and 8 μg/ml, which were intermediate. Compared with *mecA*-negative isolates, *mecA*-positive isolates showed higher resistance rates to oxacillin, penicillin, ampicillin and ciprofloxacin, but lower resistance rates to erythromycin and clindamycin (*p* < 0.05).

**Table 2:** Distribution of CoNS isolates in different MICs

No. of isolates	MICs of oxacillin				MICs of vancomycin		
	≤0.25	0.5	1	≥2	≤2	4–8	≥ 16
*mecA*-positive CoNS (*n* = 37)	0	0	0	37	25	12	0
*mecA*-negative CoNS(*n* = 65)	8	31	13	13	36	29	0

The presence of *mecA* was screened by PCR in all the CoNS isolates. As shown in Table 1, 36.3% isolates (34 isolates from bioaerosols and three from nasal swabs of farmers) were found to contain *mecA* gene. Among these *mecA*-positive CoNS isolates, 30 (two from nasal swabs of farmers) were *S. sciuri*, accounting for 71.4% of total *S. sciuri* isolates, followed by *S. equorum* (four isolates, one from nasal swab of farmer), *S. xylosus* (two isolates) and *S. gallinarum* (one isolate). In contrast, *mecA* was not found in *S. saprophyticus*, *S. haemolyticus* and *S. arlettae* isolates. The results suggested that *mecA* was wide spread in CoNS, especially *S. sciuri*, in henhouses.

### Inflammatory response induced by aerosolized MRCoNS in chickens

**Fig. 3:**
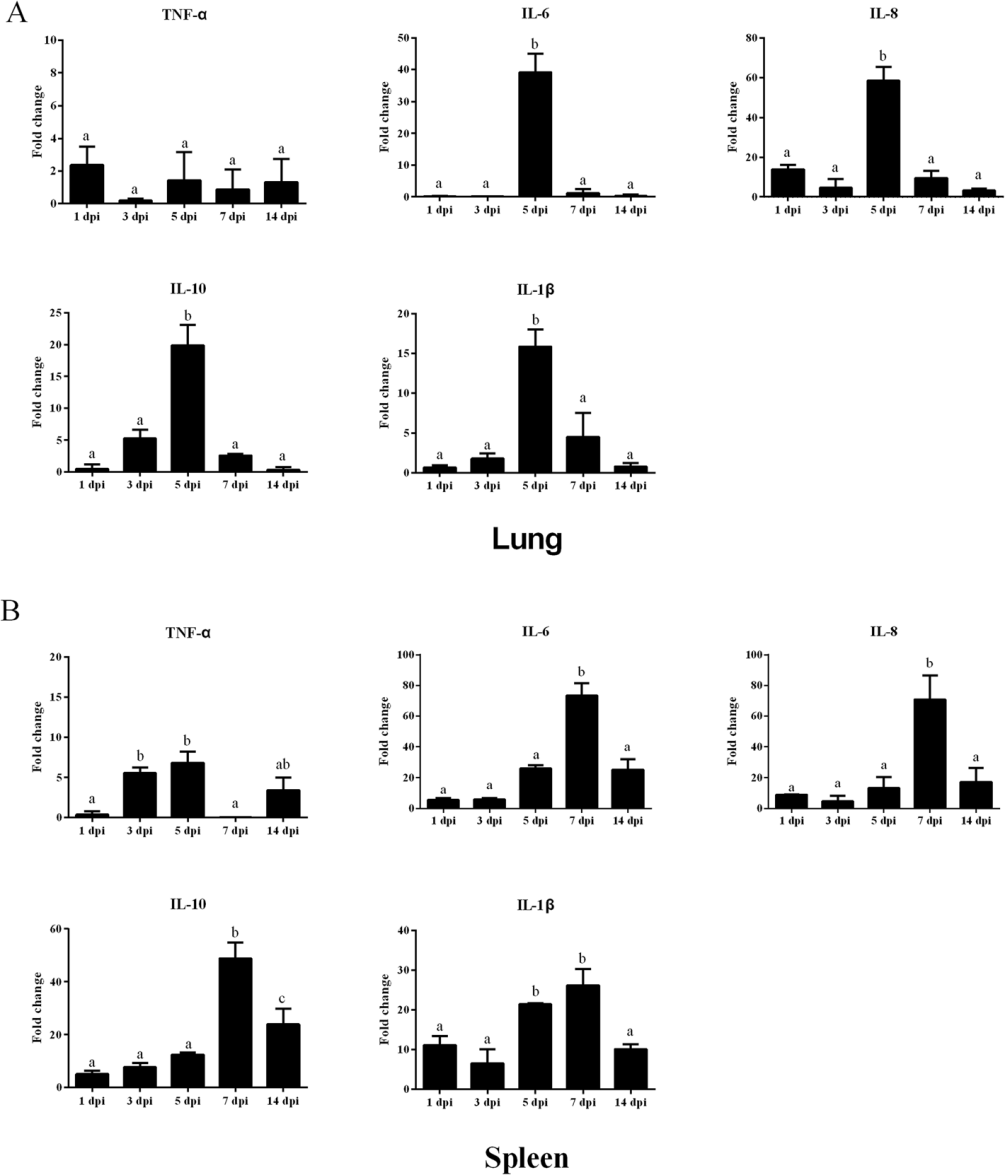
The mRNA expression of inflammatory cytokines induced by aerosolized CoNS. The different normal letters indicate significant difference among different dpi, **a**, **b** means there is no significant difference between **a**, **b** and **a**, **a**, **b** and **b**

As above described, the concentration of CoNS could reach 10^6^ magnitudes cfu/m^3^ in henhouses, so we chose approximately 1.0 × 10^6^ cfu/m^3^ as the concentration of exposure with aerosolized MRCoNS (*mecA*-positive *S sciuri*). As shown in Fig. 3, although there were no typical symptoms after infection, the expression of proinflammatory cytokines IL-1β, IL-6 and IL-8 were significantly induced at 5 dpi in lungs and at 7 dpi in spleen, and at the same time, the expression of anti-inflammatory cytokine IL-10 was also induced. Then, IL-1β, IL-6, IL-8 and IL-10 were reduced at 14 dpi. The expression of TNF-α was not greatly induced by aerosolized MRCoNS. These results suggested that aerosolized MRCoNS induced inflammatory cytokines in chickens.

## Discussion

In this study, we showed that CoNS were highly prevalent, and they were the dominant cultivable bacterial group under aerobic condition in bioaerosols in the later period of chicken breeding. In addition, CoNS were isolated from bioaerosols and farmers, and further characterized by species, antibiotic resistance, and poultry infection.

In recent decades, intensive animal production has become common, but it followed environmental concerns and public health, and bioaerosols contamination is one of the important risk factors [22]. Various bacteria have been identified in bioaerosols from animal houses, such as *Pseudomonas*, *Bacillus*, *Salmonella*, *E. coli*, *Streptococcus* and *Staphylococcus* [23]. The composition of bioaerosols in different animal houses shows different features. For example, the mean total concentration of bacteria inside swine barns was 6.6 × 10^4^ cfu/m^3^, mainly including *Staphylococcus*, *Pseudomonas* and *Bacillus* [24]. During cattle breeding, bioaerosols are the most important route of transmission for *Mycoplasma bovis* [25]. In hospitals, *Staphylococcus*, including *S. aureus* and CoNS, is one of the most serious bacteria in bioaerosols. Several studies on the bioaerosols of poultry houses have been done. In Switzerland, the mean bacterial exposure level to poultry farmers was 53 × 10^7^ cells/m^3^, and among them, 62 × 10^6^ cells/m^3^ were staphylococci [20]. In Australia and Poland, staphylococci was also one of the dominant bacteria in the poultry houses [26, 27]. Even in some broiler houses of Germany, staphylococci concentrations were higher than between 1 × 10^6^ cfu/m^3^, and affected by the wind in the barn [28]. Besides to staphylococci, potential pathogens, such as *Enterococcus* spp., *Escherichia coli* and *Salmonella* spp., were also found in poultry houses [21, 29]. However, the knowledge on the concentration and characterization of CoNS in henhouses was still limited.

We investigated the bacteria in bioaerosol began with breeding of 1 day old chicken. Compared with membrane filtration and impaction, we could see different sampling procedures had obvious influences on the results, and two samplers, the Andersen 6-stage sampler and the All glass impinger-30 (AGI-30), were recommended as standards for sampling of microbiological aerosols: [24, 30]. In this study, we chose SKC BioSampler, which is All-Glass-Impinger for sampling. Our results showed that the total bacterial counts, especially the CoNS counts, were significantly increased during breeding. Especially in the later period in our tests, 75.4% of bacteria in bioaerosols were CoNS. These results suggested that CoNS were the dominant bacterial group in the bioaerosols of henhouses. CoNS can colonize well on the surface of animal skin and have biofilm forming ability to resist environmental stresses [31], and these phenotypes help them to survive and spread in the bioaerosols. Compared with swine houses, that the overall mean total concentrations of cultivable bacteria inside the barns were 8.6 × 10^4^ cfu/m^3^ as measured by impaction [24], higher concentrations of bacteria in bioaerosols were found in chicken houses, and wing-fanning of chickens might be an important reason.

Seven species of CoNS were identified in bioaerosols and nasal swabs of farmers, and *S. sciuri* was the predominant species, which was consistent with the incidence in other environments of chicken breeding, like bedding and litter [32, 33]. Among our identified species, *S. saprophyticus* and *S. haemolyticus* were frequently recovered from humans. In the previous study, the CoNS were recovered from chicken and their carcasses, minced meat and the contact persons, suggesting its potential transmission from animal to humans [34]. Most human infections caused by CoNS are subacute and chronic [35], but sometimes, foreign-body-related infections of the bloodstream by CoNS can be fatal [36, 37]. As opportunistic pathogens, the presences of high concentrations of CoNS in bioaerosols have a potential risk of disease for chickens as well as poultry farmers.

Antibiotic resistance and resistance transmission of CoNS in bioaerosols is another important risk factor, for not only poultry industry but also public health. According to previous reports, hospital-acquired MRSA is often multidrug resistant, while community-acquired MRSA strains are usually limited to beta-lactam resistance and are susceptible to fluoroquinolones, aminoglycosides, erythromycin, and clindamycin [38, 39]. In contrast, the staphylococci from animal breeding have different resistance patterns [40]. Our CoNS isolates showed high resistance rates to penicillin, ampicillin, ciprofloxacin, chloramphenicol and tetracycline, which were used in animal breeding, suggesting the importance of selection stress from antibiotics. In addition, 49.0% CoNS isolates were MRCoNS (MIC ≥2), and among them, 74% contains *mecA*, which was responsible for its resistance. *S. aureus* is one of the most important pathogens for human and animals and widely exist in poultry breeding [41]. MRSA, which contains the horizontally transferable methicillin*-*resistance gene *mecA*, was a global risk to human health [17]. Because CoNS is a closely related staphylococci of *S. aureus*, it is considered as an important resistance determinants provider of *S. aureus*. In this study, high percentage of CoNS isolates (36.3%) contained *mecA* gene, and this is of concern of potential spread of *mecA* to *S. aureus* in the henhouses. What was more serious, lots of MRCoNS isolates were not susceptive to vancomycin, which was the gold standard for treating the infections of methicillin-resistant staphylococci [42]. Therefore, these isolates had more broad-spectrum resistance, that suggested the emergence of multi-drug resistant CoNS in the bioaerosols of poultry houses. In addition, it has been proved that bacteria, such as *E. coli* carrying plasmid-mediated quinolone resistance genes, could spread from farms to the external environment via air [43]. In this study, although we did not test the CoNS around the henhouses, we suppose that the multi-drug resistant CoNS can spread out from the inside of henhouses to the outside through the air, which further highlight its serious threat to public health.

CoNS are the predominant pathogens causing intramammary infections in dairy cows [14], while in chickens, the economic burden of staphylococcal infections includes decreased weight gain, drop in egg production, mortality, condemnation at slaughter and lameness [33]. Although obvious symptoms caused by CoNS did not occurred frequently, immunological challenge from CoNS bioaerosol was one of the important negative effects in chickens. To simulate the CoNS bioaerosols infection, we chose *mecA*-positive *S. sciuri*, which was the predominant species in the henhouses, as the model of aerosolized bacteria, and we chose 1.0 × 10^6^ cfu/m^3^, which was the detected concentration in this study, as the concentration of exposure. As shown in Fig. 3, although there were no typical symptoms caused by independent CoNS infection, proinflammatory cytokines including IL-1β, IL-6 and IL-8 were significantly induced at 5 or 7 dpi, and at the same time anti-inflammatory cytokine IL-10 was also up-regulated. Both of them were reduced at 14 dpi to maintain a homeostatic state. These results suggest that exposure in aerosolized CoNS could induce obvious inflammatory response in chickens. It is evident that the inflammatory process is a life-saving response to microbial challenge, however, it is supported by available nutrients diverted from productive purposes [44, 45]. As previously reported, equalized for feed intake, a vigorous acute phase immune response in chickens has been estimated to account for around 10% of nutrient use [46], and the threonine requirement increase by 2 to 10% [47]. A general model for predicting animal performance during pathogen challenges suggested that, subclinical infections, even with no visible symptoms but immune responses, caused a reduction in feed intake, and greater reduction was seen during clinical disease [48]. Therefore, the induction of inflammatory cytokines by CoNS suggested that, continuous exposure to high concentration of CoNS had caused subclinical infection of chickens, which could lead to reduced production performance. In addition, CoNS were also isolated in the nasal swabs of poultry farmers, which suggested that the presence of CoNS in bioaerosols might represent a significant immunological challenge to chickens as well as farmers.

## Conclusions

In this study, we showed high concentration of CoNS in henhouses, and *S. sciuri* was the preponderant CoNS species. Antibiotic resistance analysis and bioaerosols infection of CoNS further highlighted its hazards on resistance and immunological challenge. These results suggested that, CoNS in bioaerosols could be a serious factor in the henhouses for not only poultry industry but also public health.

## Methods

### Sampling from the henhouses

Bioaerosol samples were collected from nine henhouses for the growing period of caged Hy-Line Layers in Central China during April to July 2017 and 2018. The sampling times covered the first 90 days of the growing period before changing their cage (chickens age 2, 20, 40, 60 and 90 days). All the houses were strictly disinfected before breeding. The used sampler was SKC BioSampler (SKC, Pittsburgh, PA, USA) and the flow rate was 12.5 L/min. According to the indoor air quality standard in China (GB/T18883–2002), bioaerosols were collected at five sampling sites, which were evenly distributed in the henhouses. At each sampling site, the sampling time was 20 min and a total of 250 L bioaerosols were collected into 20 ml phosphate-buffered saline (PBS). The collected samples were kept in 50 ml sterile centrifuge tubes in an ice bath and then transferred to the laboratory for analysis immediately. Nasal swabs of farmers in each henhouse were also collected and transported to the laboratory for bacterial isolation.

### Enumeration, isolation and identification of staphylococci in bioaerosols

To count the total culturable bacteria and staphylococci from bioaerosols, the samples in PBS were 10-fold serial diluted, and spread onto Tryptic Soy Agar (TSA) (BD, Franklin Lakes, NJ, USA) plates and Baird Parker plates (BD) at 37 °C for 24 h. The suspect staphylococcal colonies on the Baird Parker plates were confirmed by Gram stain and PCR targeting the *Staphylococcus*-specific 16S rRNA fragment as previously described [49]. The bacterial count in each henhouse was calculated as the mean from five sampling sites. To further isolate and identify the species of staphylococci, three to four suspect colonies from each plate were picked randomly and identified using 16S rRNA sequence analysis and Microbiology Identification System Phoenix-100 (BD). To isolate and identify the staphylococci from poultry farmers, the collected nasal swabs were streaked onto Baird Parker plates and cultured at 37 °C for 24 h, and the suspect staphylococcal colonies were identified as described above.

### Antibiotic susceptibility testing

CoNS isolates were tested for susceptibility to antimicrobial drugs using disk diffusion assay or minimum inhibitory concentration (MIC) assay according to the Clinical and Laboratory Standards Institute (CLSI) [50]. Disks were placed on the surfaces of CoNS inoculated Mueller Hinton agar plates (Oxoid, Basingstoke, UK). These antimicrobial disks (Oxoid) included penicillin (Pen, 10 U), ampicillin (Amp, 10 μg), ciprofloxacin (Cip, 5 μg), chloramphenicol (Chl, 30 μg), erythromycin (Ery, 15 μg), tetracycline (Tet, 30 μg), amikacin (Ami, 30 μg), rifampicin (Rif, 5 μg) and clindamycin (Cli, 2 μg). Inoculated plates were incubated at 37 °C for 24 h. The inhibition zone diameters were measured and interpreted following the CLSI guidelines. MIC assays were carried out to determine the susceptibility of CoNS to oxacillin (used for confirmation of methicillin resistance in CoNS, CLSI) and vancomycin, and the evaluated concentrations were 0.125–128 μg/ml for oxacillin and 0.125–16 μg/ml for vancomycin. *E. coli* ATCC 25922 and *S. aureus* ATCC 25923 strains were included in the test for quality control.

### Detection of *mecA* in CoNS

Genomic DNA was extracted using MiniBEST Universal Genomic DNA Extraction Kit (TaKaRa). The presence of *mecA* was detected using PCR targeting the fragment of *mecA* (163 bp), as previously reported [51]. PCR was performed in a GeneAmp PCR System 9700 (ABI, Darmstadt, Germany). The primers were mecAF, 5′-ACTGCTATCCACCCTCAAAC-3′ and mecAR, 5′-CTGGTGAAGTTGTAATCTG-3′. The reaction conditions were as followed: initial denaturation temperature of 94 °C for 5 min, followed by 35 cycles of denaturation at 94 °C for 30 s, renaturation at 55 °C for 1 min, elongation at 72 °C for 30 s and final elongation at 72 °C for 5 min. The PCR products were subject to agarose gel electrophoresis. The DNA bands were stained with ethidium bromide and visualized using a GelDoc XR System (Bio-Rad, Shanghai, China).

### Immune responses of chickens infected with aerosolized CoNS

To investigate the effects of chickens exposed to aerosolized CoNS, bioaerosol infection was carried out. Embryonated eggs from SPF Leghorn chickens were purchased from Merial-Vital, Beijing, China. SPF chickens were hatched in a contained environment, and raised in negative pressure isolators for animal work. A total of 40 1-day-old SPF chickens were randomly divided into two groups (*n* = 20). Chickens in the infection group were exposed to bioaerosols containing approximately 10^6^ cfu/m^3^ *mecA*-positive *S. sciuri* for 1 h (range from 6 × 10^5^ to 2.5 × 10^6^ cfu/m^3^ during this hour), and were transferred into individually ventilated cages for breeding. Aerosolized bacteria were exported by Atomizer Aerosol Generator Model 3079A (TSI Incorporated, Shoreview, MN, USA) as previously reported [52], and the particle diameter was 0.2–0.3 μm. The concentration of bacteria in the aerosol (20 cm above the ground of the cages) was assessed every 15 mins using plate sedimentation method, and a proper amount of aerosolized bacteria was replenished every 15 mins. Chickens exposed in exported PBS without bacteria were used as a control group (*n* = 20). At 1, 3, 5, 7 and 14 days post infection (dpi), four chickens in each group were randomly selected, and the total mRNA of lungs and spleens was isolated using MiniBEST Universal RNA Extraction Kit (TaKaRa, Dalian, China). The expression levels of interleukin (IL)-1β, IL-6, IL-8 and tumor necrosis factor (TNF)-α were detected by real-time reverse transcription (RT)-PCR. The *gapdh* gene was used as the internal control [53]. The primers were as follows: *gapdh*-f, 5′-TCTCCATGGTGGTGAAGACA-3′, *gapdh*-r, 5′-GACGTGCAGCAGGAACACTA-3′; IL-1β-f, 5′-GGATTCTGAGCACACCACAGT-3′, IL-1β-r, 5′-TCTGGTTGATGTCGAAGATGTC-3′; IL-6-f, 5′-ATCCGGCAGATGGTGATAAA-3′, IL-6-r, 5′-CCCTCACGGTCTTCTCCATA-3′; IL-8-f, 5′-GCAAGGTAGGACGCTGGTAA-3′, IL-8-r 5′-CCAAGCACACCTCTCTTCCA-3′; TNF-α-f, 5′-CTTCTGAGGCATTTGGAAGC-3′, TNF-α-r 5′-ACTGGGCGGTCATAGAACAG-3′. Each assay was carried out with at least three biological replicates. After the experiment, the chickens were euthanized and underwent harmless treatment according to the regulations from Hubei Provincial Animal Care and Use Committee.

### Statistical analysis

To test the bacterial loads in the bioaerosols, the bacteria count in each henhouse was calculated as the mean from five sampling sites, and the bacteria counts at different times were compared using Student’s *t*-test. To evaluate the inflammatory responses induced by aerosolized CoNS, the relative transcript abundance levels of inflammatory cytokines were calculated using the 2^–ΔΔCT^ method and the expression levels at different times were compared using Student’s *t*-test. A *p*-value < 0.05 was considered statistically significant.

## Data Availability

The datasets generated and analysed during the current study are available from the corresponding author on reasonable request.

## Abbreviations

CLSI: Clinical and Laboratory Standards Institute
CoNS: Coagulase-negative staphylococci
dpi: Days post-infection
IL: Interleukin
MIC: Minimum inhibitory concentration
MRCoNS: Methicillin-resistant CoNS
MRSA: Methicillin-resistant *S. aureus*
PBS: Phosphate-buffered saline
TNF: Tumor necrosis factor
VRSA: Vancomycin-resistant *S. aureus*
